# Topographic and quantitative relationship between prostate inflammation, proliferative inflammatory atrophy and low-grade prostate intraepithelial neoplasia: A biopsy study in chronic prostatitis patients

**DOI:** 10.3892/ijo.2012.1646

**Published:** 2012-10-04

**Authors:** A. VRAL, V. MAGRI, E. MONTANARI, G. GAZZANO, V. GOURVAS, E. MARRAS, G. PERLETTI

**Affiliations:** 1 Section of Histology, Department of Basic Medical Sciences, Ghent University, Ghent, Belgium;; 2 Urology Secondary Care Clinic, Azienda Ospedaliera Istituti Clinici di Perfezionamento, Milan;; 3 Department of Urology, San Paolo Hospital/University of Milan, Milan;; 4 Division of Pathology and Cytodiagnostics, M. Mellini Hospital, Chiari, Italy;; 5 Department of Pathology, General Hospital ‘G. Gennimatas’, Thessaloniki, Greece;; 6 Biomedical Research Division, Department of Theoretical and Applied Sciences, University of Insubria, Busto A/Varese, Italy

**Keywords:** proliferative inflammatory atrophy, prostate atrophy, prostate cancer, prostatitis, prostatic intraepithelial neoplasia, prostate, inflammation

## Abstract

Inflammatory processes are important components in the pathogenesis of many human cancers. According to the ‘injury and regeneration’ model for prostate carcinogenesis, injury caused by pathogens or pro-inflammatory cytotoxic agents would trigger proliferation of prostatic glandular cells, leading to the appearance of epithelial lesions named ‘Proliferative Inflammatory Atrophy’ (PIA). Inflammatory cells infiltrating the prostate would release genotoxic reactive oxygen species, leading atrophic cells to neoplastic progression. The hypothesis pointing to PIA as risk-lesion for prostate cancer has been extensively investigated at the cellular and molecular levels, but few morphological data are available linking PIA or prostatic intraepithelial neoplasia (PIN) to inflammation or clinical prostatitis. We investigated at the morphological level 1367 prostate biopsies from 98 patients with a recent history of chronic prostatitis, and 32 patients with biopsies positive for carcinoma. Our results show that i) PIA is found more frequently in biopsy cores containing a severe or moderate inflammatory focus, compared to NON-PIA lesions (partial or cystic atrophy); ii) the PIA lesion post-atrophic hyperplasia is more frequently found in tissues showing mild or no inflammation; iii) the extent of PIA per patient correlates with the burden of moderate or severe inflammation, whereas NON-PIA lesions do not; iv) low-grade PIN is in over 90% of cases emerging from normal, non-atrophic glands and is more frequently found in biopsy cores with absent or mild inflammatory burden; v) the inverse relationship between the prevalence of low-grade PIN and the extent of PIA lesions per patient is described by a power law function, suggesting the low likelihood of the concomitant presence of these lesions in the same tissue; vi) NON-PIA lesions correlate inversely with neoplasia in patients with prostate cancer; vii) the total scores of the NIH-CPSI questionnaire correlate with both PIA and inflammation burdens at diagnosis of prostatitis but not after pharmacological intervention. These results point to a positive association between tissue inflammation, clinical prostatitis and the putative cancer risk-lesion PIA, but do not support a model whereby low-grade PIN would arise from PIA.

## Introduction

Inflammatory processes caused by pathogens or by chemical or physical agents are important components in the pathogenesis of many human cancers (1). Increasing evidence suggests that also in the prostate gland inflammation may be implicated in oncogenesis. A recent study performed on 70,000 North American men showed that a clinical history of prostatitis significantly increases the relative risk for prostate cancer (PCa) (1.3; 95% CI: 1.1–1.5), and that a longer duration of the disease may further increase this risk (2). More indirect meta-analytic evidence showed that chronic consumption of aspirin or other NSAIDs can significantly decrease the odds of prostate cancer (PCa) (0.92; 95% CI: 0.86–0.97) (3).

In recent years, extensive research efforts have been devoted to investigate the link between inflammation and PCa and to find which inflammatory lesions of the glandular epithelium might act as early forerunners of neoplastic transformation. A new ‘injury-and-regeneration’ model, linking the effects of chronic inflammation to the molecular and cellular modifications underlying the pathogenesis of prostate cancer has been advanced by De Marzo and coworkers (4). According to this model, inflammatory cells infiltrating the prostate in response to an injury caused by infection or by the action of endogenous/exogenous irritants or cytotoxins may cause the initiation of neoplastic transformation by releasing genotoxic reactive oxygen species (ROS). Tissue injury and cell loss would at the same time trigger a tumor promotion step with proliferative regeneration of the damaged epithelium, leading to the appearance, mainly in the peripheral zone of the gland, of putative cancer ‘risk’ lesions of atrophic appearance, generally referred to as ‘Proliferative Inflammatory Atrophy’ (PIA). Proliferating atrophic cells, further exposed to ROS-induced oxidative DNA damage, might subsequently progress to the *in situ* cancer precursor prostatic intraepithelial neoplasia (PIN) and, ultimately, to frank adenocarcinoma (4).

The hypothesis pointing to PIA as a risk-lesion for prostate cancer has been investigated at the preclinical, clinical, cellular and molecular levels (reviewed in ref. 5). Very recently, the presence of atrophy has been linked to advanced prostate cancer (6,7). At the morphological level, it has been shown that high-grade (HG) PIN and PCa may merge with PIA lesions, thus suggesting the existence of a continuum between these entities (8,9). However, the issue of whether these histological findings are true signs of an undergoing PIA-cancer transition is still controversial (10–14), though some experts cautiously suggest that the great genetic instability of atrophic cells makes them more vulnerable to lesions possibly leading to neoplastic transformation (15,16).

At the genetic level, it was shown that a number of hallmarks of high-grade PIN and PCa are found in PIA cells (4,17). For example, chromosomal aberrations commonly found in PCa, like 8p22 loss, 8q24 gain, 8c gain and X gain, are also detected in PIA, albeit in the form of somatic aberrations (18–21). At the molecular level, a number of proto-oncogenes, tumor-suppressors and transducers of growth/survival signals such as NKX3.1, MSR1, Ki-67, Bcl-2, p16/CDKN2, p27, p53, GSTP1 and Cox-2, were found to be upregulated/disregulated in PIA, sometimes to an extent similar or identical to high-grade PIN or PCa (reviewed in refs. 4 and 5). The increasing evidence aimed at linking PIA, inflammation and PCa extends to findings involving the role of corpora amylacea (22), the expression of the prostate tumor overexpressed-1 (PTOV1) gene in atrophy (23), the involvement of immune regulatory cells (e.g., TH17 cells) and cytokines, and the influence of a number of PCa-associated polymorphisms in the cyclooxygenase-2 gene (reviewed in refs. 17 and 24).

Despite this mounting host of molecular evidence, very scant morphological/topographic data are available linking PIA or PIN to inflammatory findings or to clinical chronic prostatitis (CP).

To increase knowledge in this area, we investigated at the morphological level 1367 prostate biopsies from 98 patients affected by chronic prostatitis (CP) and 32 patients with a history of CP and a biopsy positive for carcinoma. The aim of this study was to investigate the topographic and quantitative relationship between inflammation, proliferative inflammatory atrophy and low- or high-grade proliferative intraepithelial neoplasia.

## Materials and methods

### Biopsy material

This study was performed on biopsy specimens from patients randomly selected from a historical collection of hematoxylin and eosin-stained, fully anonymized prostate biopsies, collected in the years 2000–2010.

Patients had been subjected to prostate biopsy to exclude the presence of malignancy, in the presence of elevated total serum PSA levels (>4 ng/ml) and suspicious clinical findings.

The inclusion criterion for this retrospective study was a recent history (<3 months) of class II chronic bacterial prostatitis (CBP, 28% of total patients) or class III chronic prostatitis/chronic pelvic pain syndrome [CP/CPPS, inflammatory subtype IIIa; NIH-NIDDK classification, (25)] (72% of total patients). At diagnosis of chronic prostatitis (CP), patients were subjected to a combined pharmacological treatment protocol of 4 weeks, including antibacterial agents, α blockers and anti-inflammatory agents (26,27).

In the presence of persisting elevated PSA levels post-therapy (27), patients were subjected to transrectal biopsies directed to the peripheral zone of the gland. A total of 1367 biopsy cores were analyzed for this study. Cores containing non-prostatic tissue (rectal mucosa and accessory glands) or gland-free prostatic stroma were discarded. Biopsy cores, previously screened for cancer by a hospital pathologist, were blind-analyzed by a histopathologist with expertise in prostate lesions to detect the presence and extent of inflammatory infiltrates, focal atrophy and other prostatic lesions. In case of uncertainty or controversy, an independent pathologist from a foreign institution was consulted.

### Characterization of extent and severity of inflammatory findings

Chronic inflammation was categorized according to the consensus classification system proposed by Nickel *et al*(28). According to this system, the localization of inflammatory infiltrates can be glandular, periglandular or stromal, the extent of prostatic inflammation is classified as focal, multifocal or diffuse, and the severity of the inflammatory finding is categorized as mild, moderate or severe. To classify the severity of inflammation, we adopted as a visual reference the pictures shown in the Song *et al* prostate histologic inflammation study (Fig. 2 in ref. 29). The present study did not focus on acute inflammation.

### Classification and quantitative estimation of focal prostatic atrophy and other non-neoplastic glandular lesions

Atrophy detected in biopsy specimens was classified according to the 2006 International Working Group Classification System for Focal Prostate Atrophy Lesions (30).

**Figure 1:**
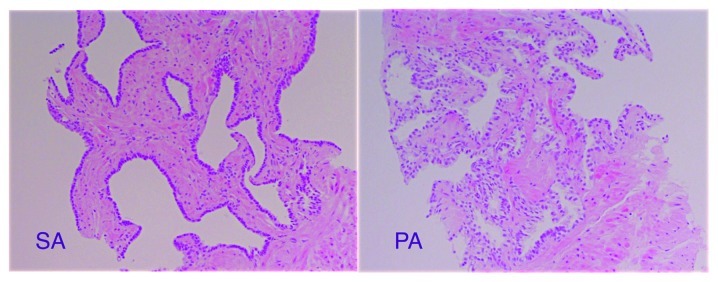
The most representative and more prevalent ‘PIA’ lesion is simple atrophy (SA, left). SA is characterized by a flattened epithelium composed of tightly-packed cuboidal cells, sometimes containing mitotic figures, in medium to large-sized glands lacking papillary infoldings. Single cells and the epithelium as a whole show a dark appearance caused by a high nucleus/cytoplasm ratio. The most representative ‘NON-PIA’ lesion is partial atrophy (PA, right), consisting of cells with pale cytoplasm, scant at cell apex but rather abundant at the side of nuclei. Nuclei are roundish or spindle-shaped. Glands are small to medium in size; although the clear-appearing epithelium may be wavy in appearance, it lacks true papillary infoldings.

This consensus system recognizes four categories of focal atrophy: simple atrophy (SA), post-atrophic hyperplasia (PAH), partial atrophy (PA) and simple atrophy with cyst formation (SACF). The term: Proliferative Inflammatory Atrophy (PIA) includes only two lesions: SA and PAH (30). In the present study, PA and SACF were categorized as ‘NON-PIA’ (Fig. 1).

The extent of prostatic atrophy in each biopsy was quantitatively expressed as the percentage of extent of atrophic glands over total glands, according to Billis *et al*(31). From these values, a ‘PIA index’, i.e., the total percentage of PIA over total glands per-prostate (i.e., per patient) was calculated.

When present, high- and low-grade PIN, basal cell hyperplasia (BCH), adenosis and atypical small acinar proliferation (ASAP) were identified and categorized according to published descriptors (32,33).

### Clinical findings

The symptoms of clinical chronic prostatitis were scored using the international, validated questionnaire National Institutes of Health Chronic Prostatitis Symptom Index (NIH-CPSI) (34). Due to the retrospective nature of this study, the questionnaire was only administered to a fraction of patients at diagnosis of prostatitis and at the end of pharmacological therapy (time of collection: 2–4 weeks after therapy). Questionnaires were fully anonymized. At the time of questionnaire administration, all patients had given their written informed consent to handling and publication of their anonymized clinical data, for scientific purposes.

### Statistical analysis

The quantitative relationship between inflammation and atrophy, inflammation and PIN, atrophy and PIN, inflammation and BCH, and atrophy and BCH was analyzed calculating the Pearson’s product-moment correlation coefficient. The XLStatistics 5.71 program (http://www.deakin.edu.au/~rodneyc/XLStatistics/) was used for analysis of data. Linear regressions, equation-finding and curve-fitting procedures were performed using the curve-fitting tool in Apple iWork Numbers ’09, version 2.1.

To analyze contingency tables containing the proportion of PIA vs. non-PIA lesions in inflammatory vs. non-inflammatory biopsy cores, we calculated the chi-square and the probability of a null hypothesis of equal proportions using the Vassar College (USA) on-line tool (http://vassarstats.net/newcs.html). To correct the assumption of continuity in the χ^2^ distribution of frequencies in 2×2 tables, we adopted the Yates’s modification of Pearson’s χ^2^ formula (35).

## Results

Equivalent proportions severe/moderate (50.28%) or mild/absent (49.72%) inflammatory infiltrates were detected in the biopsy cores analyzed in the present study. Similar numbers of atrophic lesions, dichotomized as PIA (n=840, 50.79%) or NON-PIA (n=814, 49.21%), were detected in prostate biopsies. Due to the equivalent proportions of PIA vs. NON-PIA findings, and of severe/moderate vs. absent/mild inflammation, normalization was not deemed as necessary. Low-grade and high-grade PIN were detected in total 307 and 6 biopsies, respectively.

### Relationship between inflammatory infiltrates and atrophic lesions

In order to investigate the presence and prevalence of focal atrophy in relationship to the presence and severity of inflammation, we calculated the number of biopsy cores containing i) a severe/moderate or ii) a mild or absent inflammatory infiltrate adjacent to i) a PIA (SA or PAH) or ii) a NON-PIA lesion (PA or SACF). An example of representative PIA and NON-PIA lesions is shown in Fig. 1.

**Figure 2:**
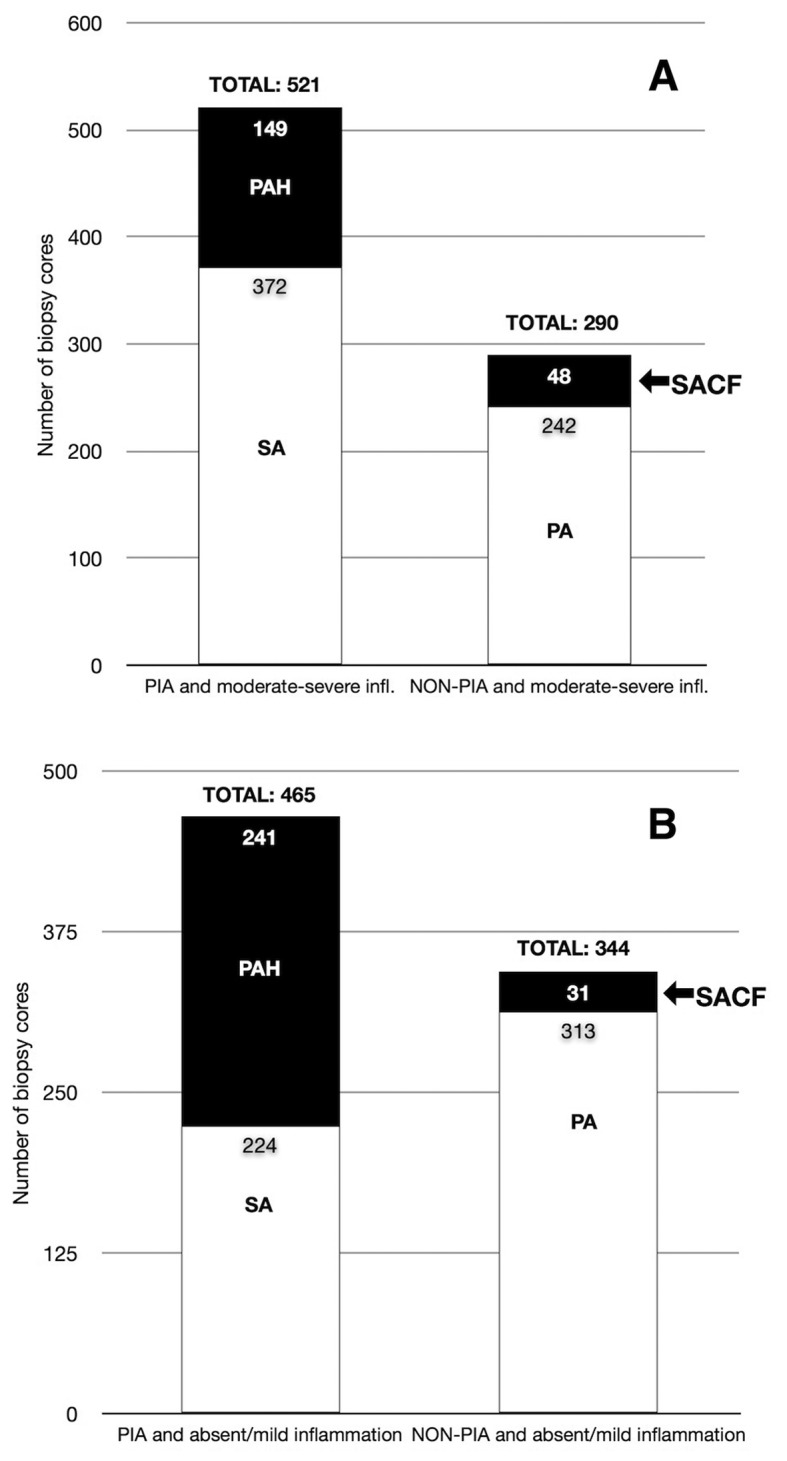
Total number of biopsy cores containing PIA or NON-PIA lesions adjacent to a severe or moderate inflammatory focus (A) or contained within a mild- or non-inflammatory specimen (B). PIA and NON-PIA lesions are also separated into the 4 categories: SA, PAH, PA and SACF.

PIA was found more frequently in cores containing a severe/moderate inflammatory finding (n=521 cores), compared to NON-PIA (n=290 cores, Fig. 2A). χ^2^ analysis rejected the null hypothesis of equal distributions of PIA vs. NON-PIA in cores harboring severe/moderate vs. absent/mild inflammation (χ^2^=7.5, P=0.0062).

When PIA and NON-PIA classes were dissected into single atrophic lesions, simple atrophy was found more frequently in severe/moderate inflammatory cores (n=372, 62%), than in cores with absent or mild inflammation (n=224, 38%) (Fig. 2). Conversely, partial atrophy was found more frequently in non-inflammatory or mildly-inflammatory cores (n=313, 56%), and to a lesser extent in cores containing a severe or moderate inflammatory focus (n=242, 44%) (Fig. 2). Interestingly, post-atrophic hyperplasia, a PIA subtype, was found to be more prevalent in tissues with mild or absent inflammation (n=241, 62%) than in inflammatory cores (n=149, 38%) (Fig. 2).

**Figure 3:**
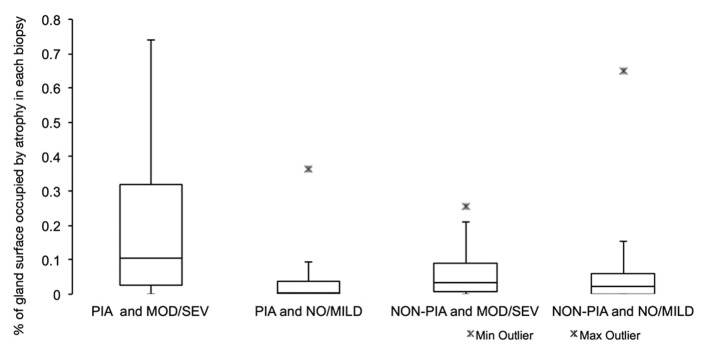
Box-and-whisker diagram comparing the the severity of inflammation [severe and moderate (MOD/SEV), vs. absent or mild (NO/MILD)] and the extent of focal atrophy (PIA or NON-PIA) in each biopsy core. On the vertical axis the the percentage of extent of atrophic glands over normal glands measured for each biopsy core is shown. The boxes define 25th and 75th percentiles, and contain the median bar. The ends of the whiskers show the lowest or highest data within 1.5 interquartile range of the lower or upper quartiles, respectively.

The box-and-whisker diagram in Fig. 3 shows the relationship between the severity of inflammation and the extent of focal atrophy (PIA or NON-PIA) in each biopsy core. Compared to non-inflammatory cores, PIA lesions were found to be more extensive in the presence of severe or moderate inflammation, whereas NON-PIA lesions were found to be equally distributed in affected prostates, irrespective of the severity of inflammation.

Besides being studied at the level of single biopsy cores, the relationship between atrophy and the severity of inflammation was investigated at the level of the whole prostate gland by calculating the percentage of glandular atrophic epithelium per-patient. If focal lesions were SA or PAH, this entity was called ‘PIA index’, whereas it was denominated ‘NON-PIA index’ when atrophy subtypes were SACF or PA.

**Figure 4:**
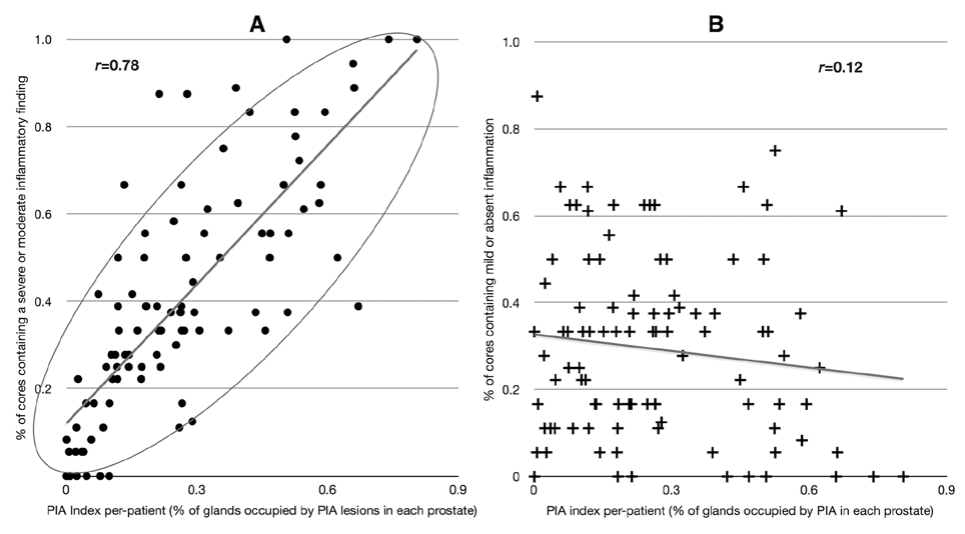

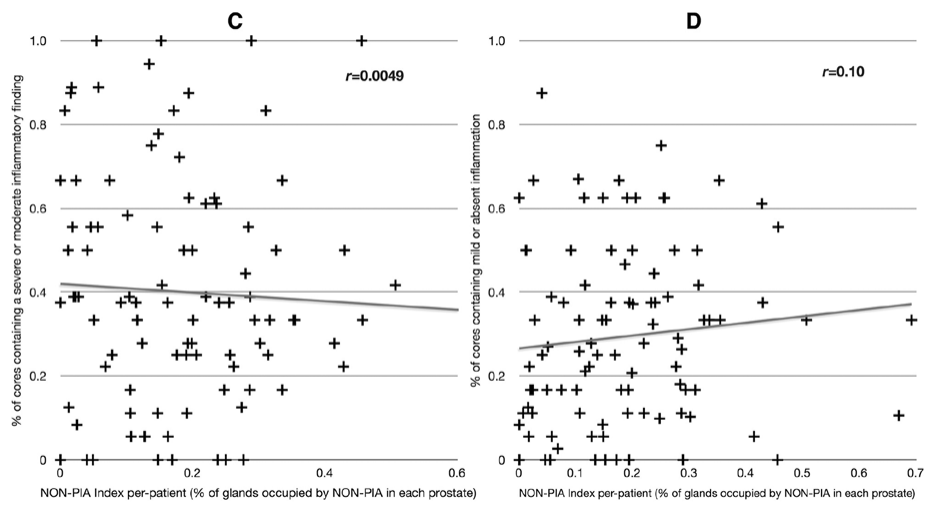
Correlation between the PIA index per-patient (i.e., the total percentage of glands occupied by PIA lesions in each prostate) and the percent of biopsy cores per patient (viz., per prostate) containing at least one severe or moderate inflammatory focus (A) or mild or no inflammation (B). In (C) and (D) a similar relationship is presented, but the NON-PIA index is plotted in horizontal axes. The Pearson’s correlation coefficient is shown in each graph.

High correlation was found between the PIA index and the percentage of biopsy cores per-patient containing at least one severe or moderate inflammatory focus (Pearson’s *r*= 0.78; Fig. 4A). Conversely, poor correlation was found when the PIA-index was compared to the percentage of biopsy cores per patient devoid of inflammation, or containing at least one mild inflammatory focus (Fig. 4B). No correlation was found between the NON-PIA index and the percentage of biopsy cores per patient containing at least one inflammatory focus of any severity (Fig. 4C and D).

### Relationship between low-grade PIN, inflammation and atrophy

**Figure 5:**
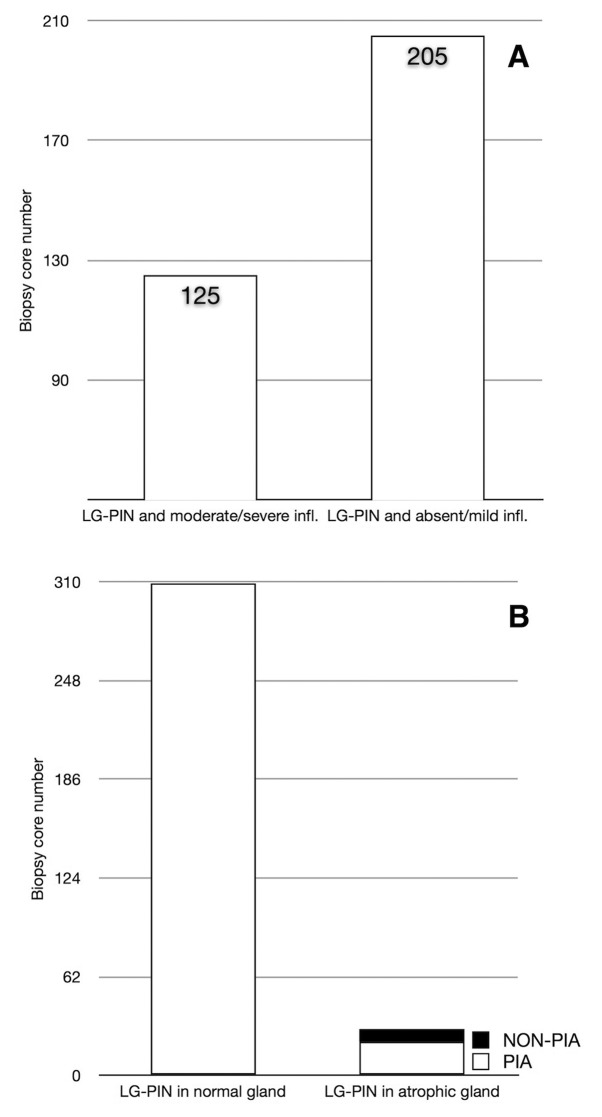
(A) Total number of biopsy cores containing at least one low-grade PIN lesion adjacent to a severe or moderate inflammatory focus (left bar), or adjacent to a mild focus or inflammation, or present within a non-inflammatory specimen (right bar). (B) Shows the total number of biopsy cores containing a low-grade PIN lesion arising from a glandular epithelium of normal appearance (left bar, n=309), or from an atrophic lesion (right bar, n=29).

To investigate at the morphological level the linkage between low-grade PIN and inflammation, we calculated the number of biopsy cores containing i) a severe/moderate or ii) a mild or absent inflammatory infiltrate adjacent to at least one low-grade PIN lesion. Low-grade PIN was found more frequently in cores devoid of inflammation, or with evidence of mild inflammation (n=205, 63%), compared to cores harboring a moderate or severe inflammatory focus (n=125, 37%, Fig. 5A).

Low-grade PIN often appears as a tufting growth of proliferating cells stemming from the lumen of prostate secretory glands or ducts. In some cases, the lesion extends to an entire gland. To investigate whether a link existed between atrophy and low-grade PIN, we analyzed the phenotype of the glandular epithelium from which PIN lesions were stemming. Three hundred and thirty-eight biopsy cores contained at least one low-grade PIN lesion. In 309 cases low-grade PIN was found to stem from a glandular epithelium of normal appearance, whereas in only 29 cases PIN emerged from, or merged with, an atrophic gland (Fig. 5B).

**Figure 6:**
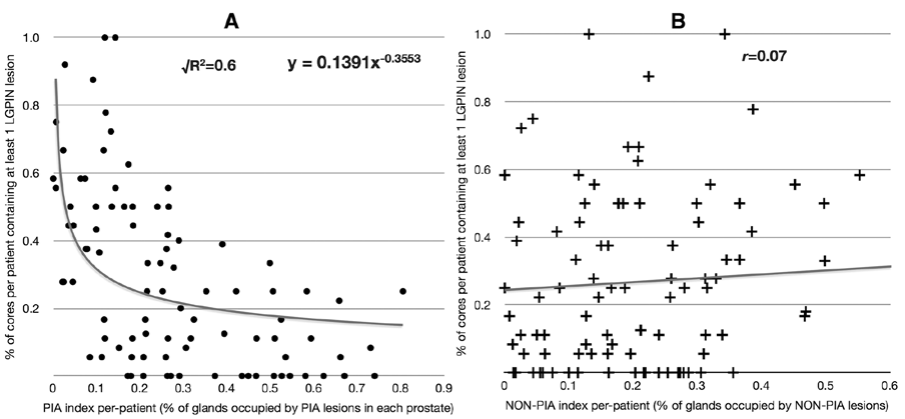
Correlation between the PIA index (A) or NON-PIA index (B) per patient (i.e., the total percentage of glands occupied by PIA or NON-PIA lesions in each prostate) and the percent of biopsy cores per patient (viz., per prostate) containing at least one low-grade PIN lesion. The equation and the square-root of the R^2^ coefficient of determination for the power law curve are given in (A). The Pearson’s *r* for the linear correlation is given in (B).

When the PIA index was plotted against the percentage of biopsy cores per patient showing at least one low-grade PIN lesion, an inverse relationship was found. This correlation was best fitted by a power decay curve (√R^2^=0.6) (Fig. 6A). Interestingly, no correlation was found between the NON-PIA index and the prevalence of low-grade PIN (Fig. 6B).

### Relationship between basal cell hyperplasia, inflammation and atrophy

Poor correlation was found between the extent of basal cell hyperplasia and inflammatory findings of any degree of severity (% cores per patient with BCH vs. % cores with severe/moderate inflammation: *r*=0.22; vs. % cores with absent/mild inflammation: *r*=0.12; graphs not shown).

Poor correlation was found between the extent of basal cell hyperplasia and the extent of focal atrophy of any kind (PIA or NON-PIA) (% cores per patient with BCH vs. PIA index: *r*=0.21; BCH vs. NON-PIA index: *r*=0.21; graphs not shown).

Although additional prostate lesions like high-grade PIN and ASAP were identified, they were not further investigated because of the small number of cases. It is important to mention that we did not observe high-grade PIN stemming from PIA lesions.

### Relationship between atrophy and prostate cancer

In 32 patients, prostate cancer was diagnosed in biopsy specimens. The same specimens were subsequently screened for focal atrophy, low- and high-grade PIN, BCH and ASAP. In these biopsies, we investigated the relationship between the extent of PCa and the extent of PIA, NON-PIA and low-grade PIN.

**Figure 7:**
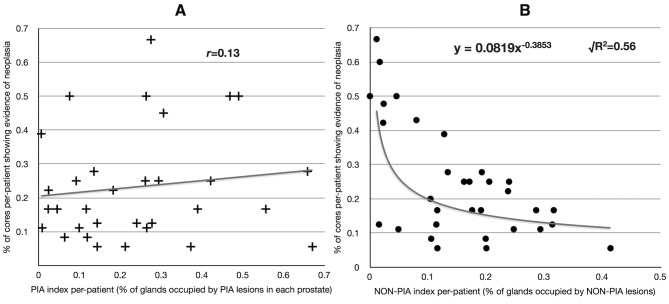
Correlation between the PIA index (A) or NON-PIA index (B) per patient (i.e., the total percentage of glands occupied by PIA or NON-PIA lesions in each prostate) and the percent of biopsy cores per patient (viz., per prostate) containing evidence of neoplasia (any Gleason grade). The Pearson’s *r* for the linear correlation is given in (A). The equation and the square-root of the R^2^ coefficient of determination for the power law curve are given in (B).

The NON-PIA index was found to be inversely related to the percent of biopsies per patient showing evidence of carcinoma. The correlation was best fitted by a power decay curve (√R^2^= 0.56; Fig. 7). In contrast, little correlation was found between the percent of biopsies showing evidence of prostate cancer and the PIA index per patient (*r*=0.13; Fig. 7).

Little correlation was found between the prostate cancer burden per patient and the percent of biopsies showing at least one low-grade PIN lesion (*r*=0.34, graph not shown).

### Relationship between atrophy, inflammatory findings and clinical symptoms of chronic prostatitis

A cohort of 34 CP patients included in the present study filled the NIH-CPSI symptom questionnaire both at diagnosis of prostatitis and at the end of pharmacological therapy. Prostate biopsies were taken 3–6 weeks after the end of therapy.

**Figure 8:**
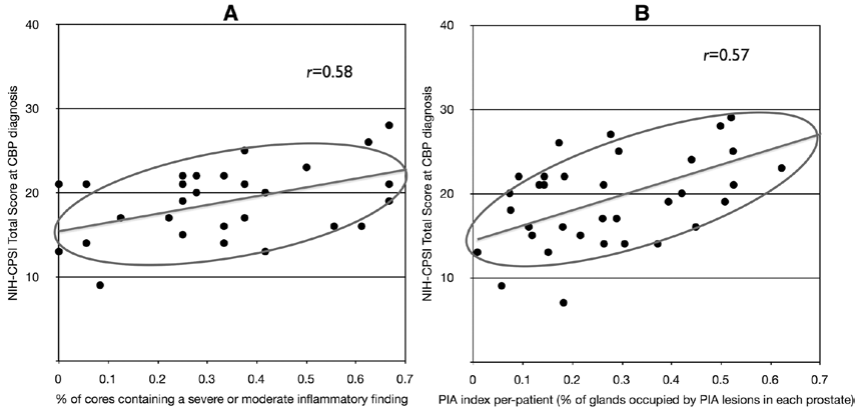
(A) Correlation between the extent of inflammation per patient, expressed as the percent of biopsy cores per-prostate containing at least one severe or moderate inflammatory finding and the total score of the NIH-CPSI questionnaire before pharmacological therapy. (B) Correlation between the PIA-index per patient, expressed as the the percentage of glands occupied by PIA lesions in each prostate and the total score of the NIH-CPSI questionnaire before pharmacological therapy. The Pearson’s correlation coefficient is shown in each graph.

When the total score of the NIH-CPSI test was plotted against the percent of biopsies per patient showing at least one moderate or severe inflammatory focus, a positive correlation (*r*= 0.58) between clinical symptoms and histologic inflam mation was found at diagnosis of chronic prostatitis (Fig. 8A), but not at the end of therapy (*r*= 0.05, graph not shown). No correlation was found between NIH-CPSI scores and mildly- or non-inflammatory findings lesions either before (*r*= 0.08) or after the end of therapy (*r*= 0.03; graphs not shown).

Similarly, When the PIA burden per patient was compared with clinical symptoms, a positive correlation (*r*= 0.57) between the total score of the NIH-CPSI test and the PIA index was found at diagnosis of chronic prostatitis (i.e., before therapy, Fig. 8B), but not at the end of therapy (*r*= 0.11, graph not shown). No correlation was found between the NIH-CPSI score and the NON-PIA index either before (*r*=0.16) or after the end of therapy (*r*=0.10; graphs not shown).

## Discussion

Since its first definition, PIA has been considered as a lesion associated with, or caused by, inflammation. However, to the best of our knowledge, very few quantitative histological-clinical studies have been conducted to support this established empirical observation. The Billis and Magna and the Ruska *et al* studies are among the few published reports investigating the relationship between focal atrophy and prostate inflammation in autopsy or biopsy material (36,37). However, both studies focused exclusively on PIA and did not include NON-PIA lesions. Billis and Magna found that PIA was associated with inflammation in 66% of analyzed prostates, whereas in 22% of cases it was not (36). In our biopsy series simple atrophy, a PIA lesion, was associated with severe/moderate inflammation in 62.4% of biopsy cores (372/596 cores, Fig. 2), whereas in the remaining 37.6% of cases contiguity with inflammatory focuses was not evident. It must be stressed that we cannot exclude that in these latter cases an inflammatory focus might have been present in adjacent tissues, not included in the biopsy core. This is an important limitation of a biopsy study.

In contrast to the Billis and Magna study, in which PAH was exclusively associated with inflammation, we found that this atrophic lesion is more prevalent in biopsy cores with mild or no inflammation (63% of cases), and less frequently present in frank inflammatory specimens (37% of cores with severe/moderate inflammation) (Fig. 2). This latter percentage is strikingly similar to the fraction of PAH associated with moderate/severe chronic inflammation (34%) reported in the Ruska *et al* biopsy study (37). Thus, despite its inclusion into the ‘PIA’ category, PAH may appear to be in most cases a non-inflammatory lesion. Indeed, as stated by McNeal (38) and according to our experience, PAH may represent a ‘post-inflammatory’ atrophic lesion. This definition is supported by the morphological evidence that in PAH the glandular lumen often comprises cell-free areas [defined by Srigley as the ‘atrophic’ component of PAH, (39)], likely constituting the remnants of a previous inflammatory injury. Proliferative regeneration of secretory cells following disruption of the epithelium, may generate small, hyperplastic glands arranged in a lobular configuration, the typical feature of PAH. In our opinion, the fact that in over 60% of cases PAH was not associ ated with a moderate or severe inflammatory focus, is not sufficient to rule out the essential inflammatory nature of this lesion.

In the present study, partial atrophy, the most representative NON-PIA lesion, was found to be in a slight majority of cases associated with mild or no inflammation (56% of cores; Fig. 2). In our opinion, the fact that PA was found to be adjacent to moderate/severe inflammatory foci in 44% of analyzed cores should not be overlooked. Recently, Billis and coworkers have demonstrated that a mergence between PA and SA occurred in 27% of prostatic biopsies, with transitions often taking place within the same gland (40). The authors hypothesized that PA may be part of an evolving ‘continuum’ in focal prostatic atrophy, with SA arising from PA in this morphologic sequence. However, in no case did the authors find inflammatory cells in biopsy cores containing PA. Because of this finding, Billis and coworkers hypothesized that inflammation might represent a secondary phenomenon in complete focal atrophy. In contrast with these findings, in our study PA could be found adjacent to moderate or severe inflammation in more than 40% of cases, although in 217 cases out of 242 inflammation was classified as ‘moderate’ and in the remaining cases as ‘severe’. Thus, our data may in part support the hypothesis that PA could precede SA, as the result of a phenotypic sequential evolution, triggered by inflammation. This is supported by the fact that, like Billis and coworkers, we observed occasional PA-SA transitions within the same gland (data not shown). However, in contrast to Billis and coworkers, we maintain the view that inflammation is more likely to be a *primum movens* in the development of PIA, rather than a mere secondary phenomenon. A study is in progress to verify the ‘continuum’ hypothesis in patients subjected to multiple biopsies and/or radical prostatectomy.

An interesting finding in our study was the dramatic decline of the burden of low-grade PIN lesions, concomitant with the increase of the extent of PIA in the same gland. This inverse relation was best fitted by a power function decay curve (Fig. 6). This finding suggests that LG-PIN and PIA may be mutually exclusive lesions within the same tissue or tissue area, and it is supported by our finding that in about 95% of cases LG-PIN arises from a normal rather than from an atrophic gland (Fig. 5). In 2009, De Marzo and coworkers promoted their ‘injury-and-regeneration’ model for prostate carcinogenesis in the frame of a comprehensive review article (4). In that context, the authors included low-grade PIN as a possible intermediate lesion, developing from PIA, and representing a putative - although non-obligate - forerunner of high-grade PIN or cancer (Fig. 3 in ref. 4). The results of the present study seem to exclude this hypothesis, also given that low-grade PIN is more frequently found in non-inflammatory tissues (Fig. 5), which also seems incompatible with the higher prevalence of PIA adjacent to focuses of severe or moderate inflammation.

In the present study we failed to demonstrate a positive correlation between the extent of PIA lesions and the per patient burden of prostate cancer (Fig. 7). Intriguingly, prostate cancer and NON-PIA lesions tend to be mutually exclusive in the same tissue or tissue area (Fig. 7). This finding, to be confirmed in a larger sample of patients, weakens the hypothesis of a possible linkage between cancer and NON-PIA lesions. Notwithstanding this evidence, if a ‘continuum’ has to be hypothesized between partial atrophy, PIA and cancer, the former and the latter lesions are most likely to occur far apart in time.

Another interesting finding of the present study is the good correlation [Portney and Watkins criteria, (41)] between the total NIH-CPSI symptom score and the extent of both inflammation and PIA at diagnosis of chronic prostatitis (Fig. 8). This is to our knowledge the first study attempting to link the clinical symptoms of CP to the extent and severity of histological prostate inflammation, as well as to the extent of focal atrophy. The absence of a correlation between these entities at the end of therapy (few days before biopsy) is probably due to the attenuation of clinical symptoms of CP achieved by aggressive pharmacological therapy, and may indirectly support the link between inflammation and symptom severity during the active (inflammatory) phase of CP. Although the small sample size (n=34) does not allow to draw a conclusive answer, the correlation between NIH-CPSI scores and inflammation is indicative of a relationship between the clinical and histological aspects of chronic prostate inflammation. Whereas such a relationship can be expected in patients suffering from inflammatory subtypes of chronic prostatitis (class II CBP and class IIIa CP/CPPS), the correlation between symptom severity and the per patient extent of atrophy is more striking, and supports the hypothesis that an inflammatory injury caused by infection or other etiological determinants may be the original causative factor of the atrophic process (4).

In conclusion, the evidence emerging from the present study i) points to a positive association between tissue inflammation and PIA, ii) questions the presumed non-inflammatory nature of partial atrophy and iii) does not seem to support a model whereby low-grade PIN would arise from PIA lesions.
